# Supplementary figures and images for: Phanerozoic radiation of ammonia oxidizing bacteria
Source: Sci Rep. 2021 Jan 22;11:2070. doi: 10.1038/s41598-021-81718-2 (PMC7822890; doi:10.1038/s41598-021-81718-2)

Tree scale: 1

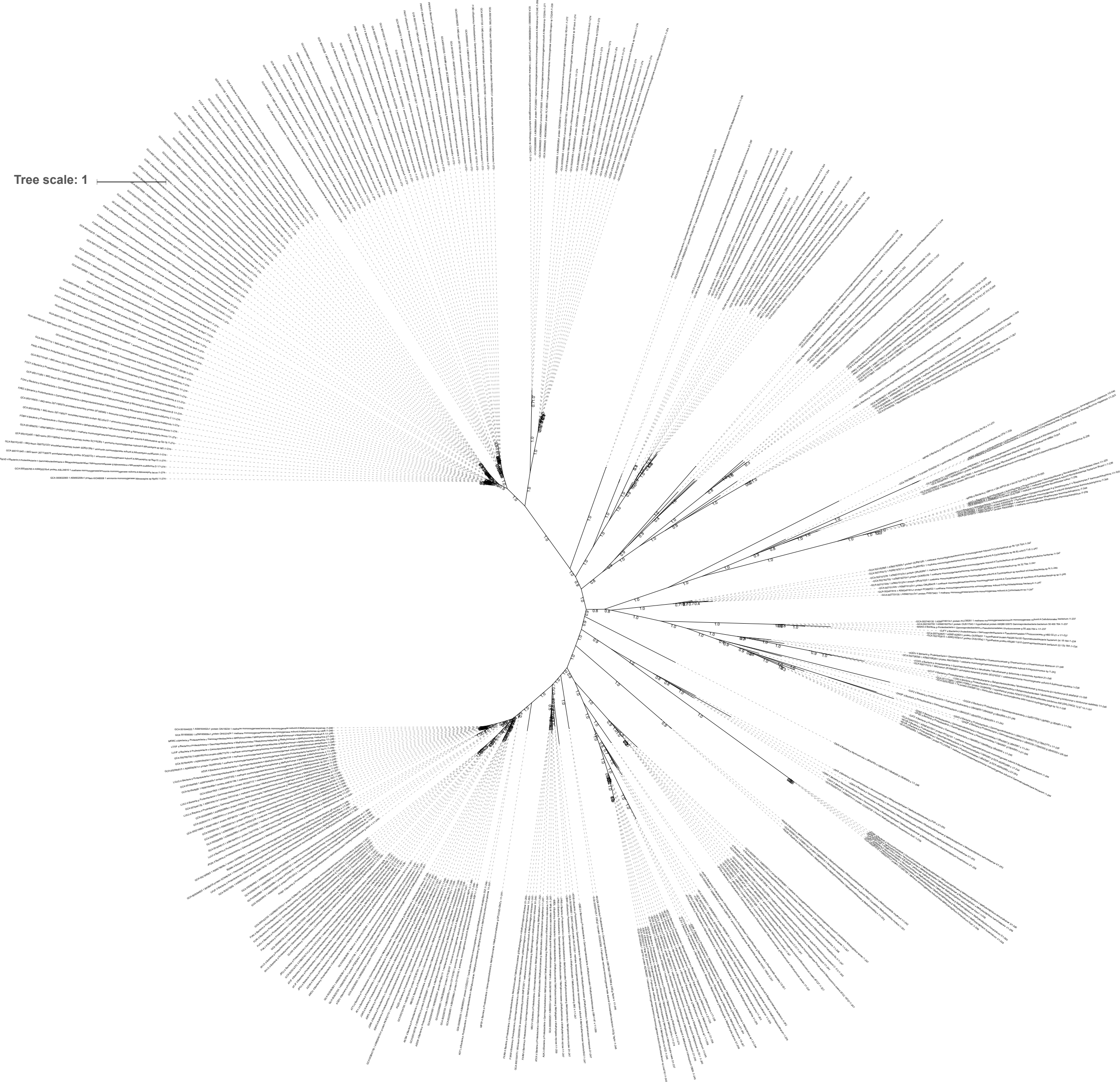

Supplement: Supplementary file 2 — Supplementary Figure 1. [file 41598_2021_81718_MOESM2_ESM.pdf]

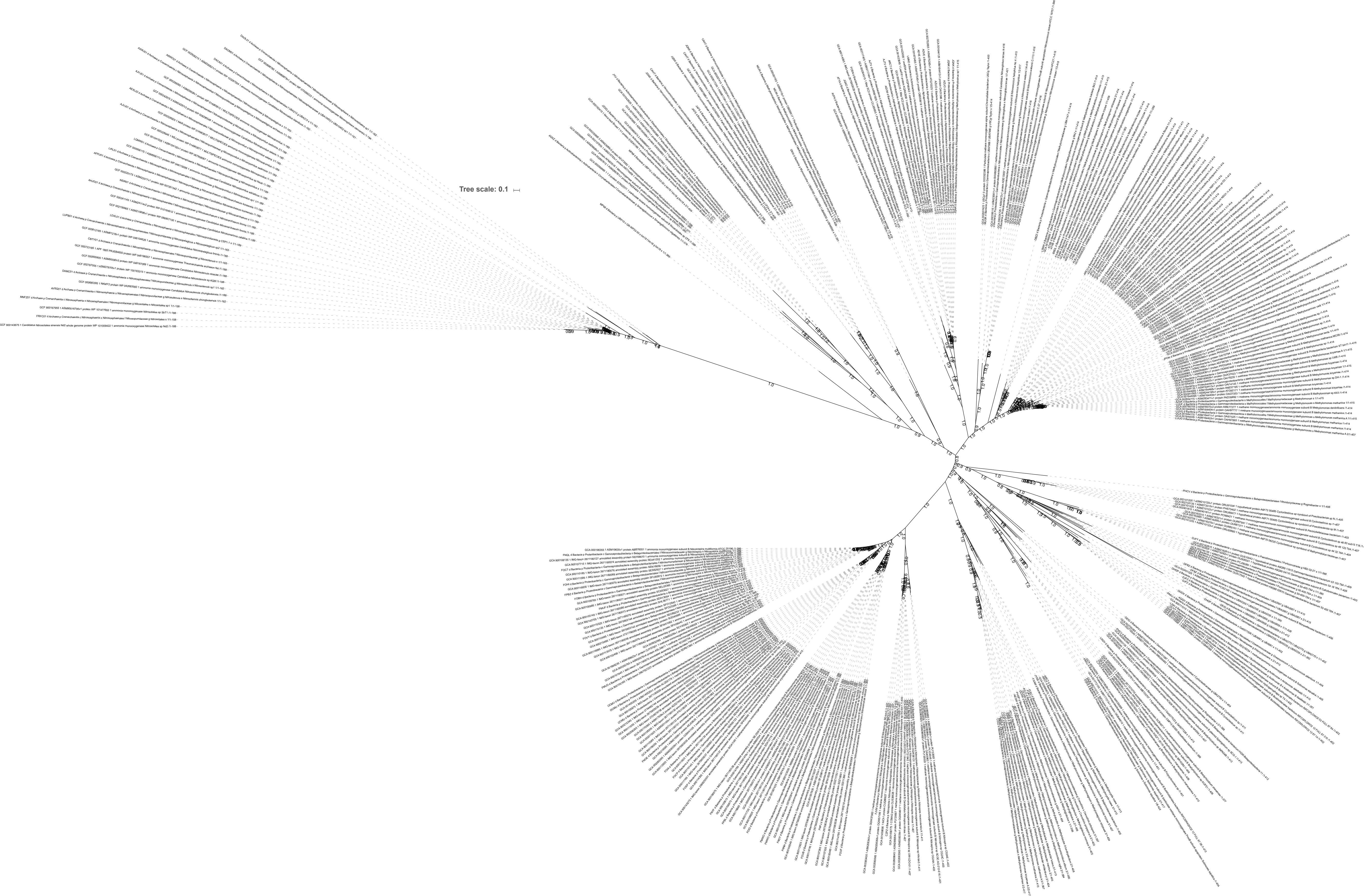

Supplement: Supplementary file 3 — Supplementary Figure 2. [file 41598_2021_81718_MOESM3_ESM.pdf]

Tree scale: 1

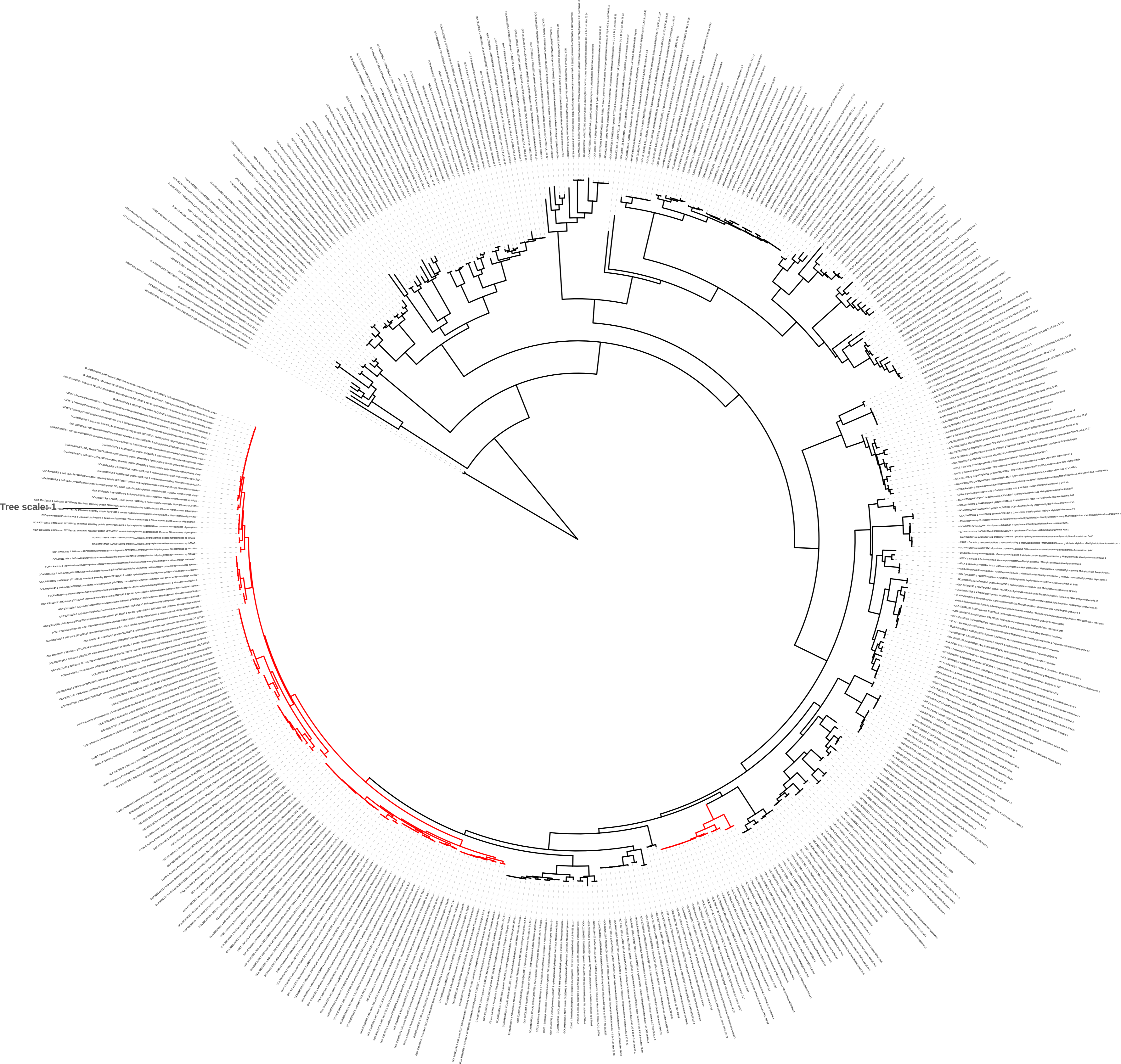

Supplement: Supplementary file 4 — Supplementary Figure 4. [file 41598_2021_81718_MOESM4_ESM.pdf]

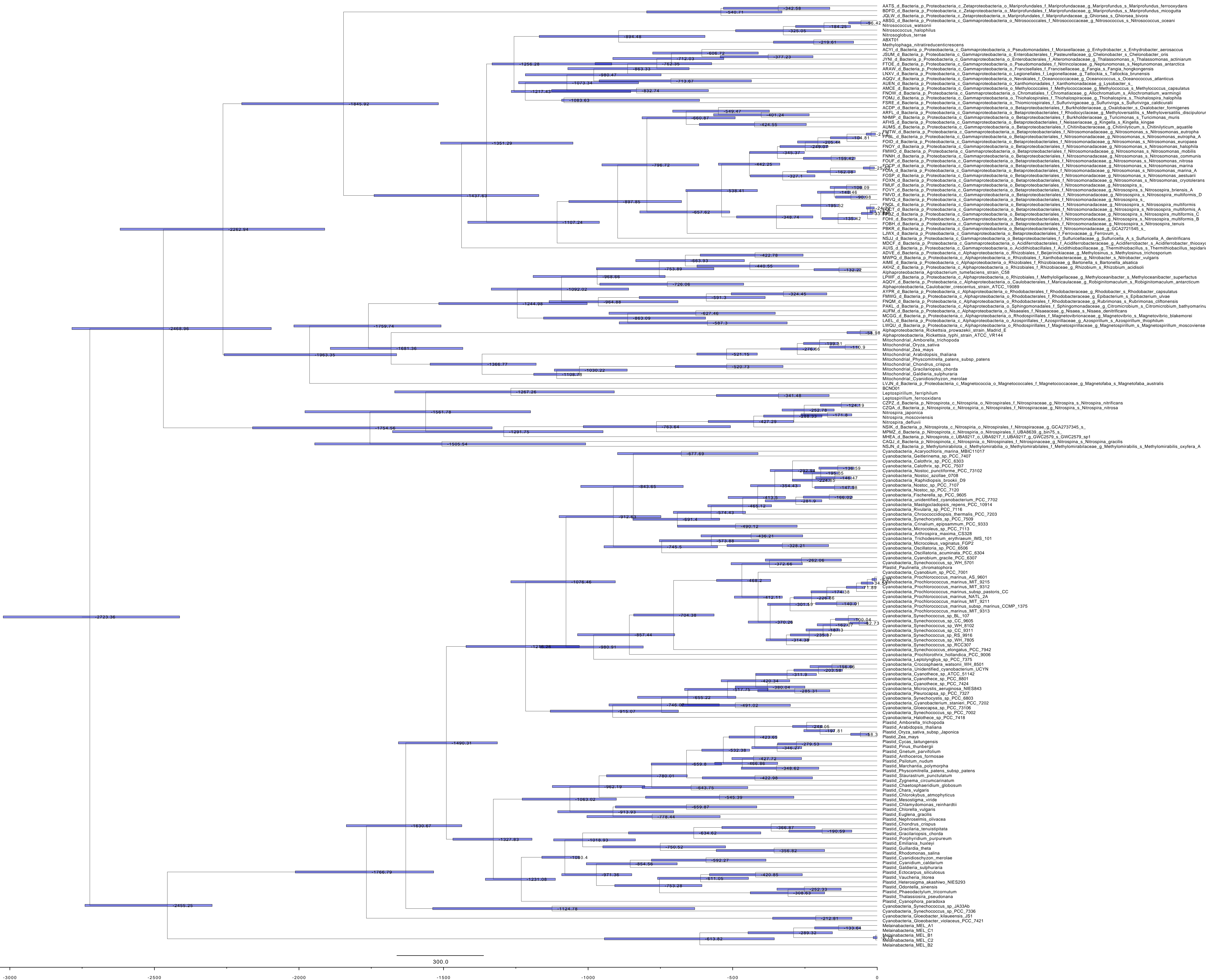

Supplement: Supplementary file 5 — Supplementary Figure 5. [file 41598_2021_81718_MOESM5_ESM.pdf]
